# Rigidez Arterial e Comprometimento Funcional na Síndrome Pós-COVID-19: Um Enigma Multifatorial

**DOI:** 10.36660/abc.20260519

**Published:** 2026-07-30

**Authors:** Mario Fritsch Neves, Michelle Rabello Cunha

**Affiliations:** 1 Universidade do Estado do Rio de Janeiro Rio de Janeiro RJ Brasil Universidade do Estado do Rio de Janeiro, Rio de Janeiro, RJ – Brasil

**Keywords:** Rigidez Vascular, Síndrome de Pós-COVID-19 Aguda

O estudo transversal apresentado no manuscrito "Rigidez Arterial, Desfechos Clínico-Funcionais e Qualidade de Vida em Pacientes Pós-COVID-19: Um Estudo Transversal" oferece uma contribuição ao entendimento das sequelas persistentes da infecção por SARS-CoV-2.^1^ Os autores demonstram que indivíduos no período pós-COVID-19, em comparação a controles saudáveis, apresentam maiores valores de velocidade de onda de pulso (VOP), principal marcador de rigidez arterial. Também evidenciam um maior índice de incremento (AIx@75), que não indica diretamente rigidez arterial, mas é um parâmetro de reflexão da onda pulso, que pode ser influenciado pela rigidez arterial, mas também por outros fatores de confusão. Esses achados alinham-se com evidências globais, como o estudo multicêntrico CARTESIAN, que identificou um envelhecimento vascular precoce em sobreviventes da doença, com um impacto particularmente pronunciado em mulheres.^2^

Um aspecto intrigante do manuscrito é a observação de que, embora a VOP esteja elevada no grupo pós-COVID, a análise ajustada sugere que esse aumento foi determinado predominantemente pela idade e pela pressão arterial sistólica, e não pela condição pós-COVID-19 de forma independente. Por outro lado, alguns estudos indicam que o efeito deletério do vírus nos marcadores de rigidez arterial e função endotelial pode persistir por meses após a resolução da fase aguda.^3,4^

Além das alterações vasculares, o comprometimento funcional e a redução na qualidade de vida emergem como pilares da síndrome pós-COVID. O manuscrito relata pior desempenho no teste de sentar-se e levantar, níveis elevados de fadiga e deterioração da saúde física e mental. Tais resultados são corroborados por pesquisas que mostram que mesmo pacientes com casos leves a moderados sofrem reduções clinicamente relevantes na qualidade de vida, muitas vezes comparáveis a pacientes com doença pulmonar obstrutiva crônica ou fibromialgia. Também destacam que a gravidade da fadiga é um preditor central das limitações na vida cotidiana desses indivíduos.^5^

O mecanismo por trás desse declínio funcional parece ser multifatorial e não restrito apenas à macrovasculatura. Hiperatividade simpática muscular e estresse oxidativo podem persistir até dois anos após a infecção.^3,4^ Essa hiperatividade autonômica, associada à disfunção microvascular e à inflamação sistêmica persistente, pode comprometer o transporte de oxigênio para os músculos esqueléticos, explicando a intolerância ao exercício observada no presente manuscrito.

A síndrome pós-COVID-19 não é apenas um evento cardiovascular, mas um estado de hipercatabolismo e fragilidade nutricional que alimenta um ciclo vicioso de declínio funcional. Somando-se à complexidade vascular e autonômica, evidências recentes destacam que o comprometimento funcional é profundamente influenciado por alterações na composição corporal e deficiências nutricionais. A desnutrição emerge tanto como fator de risco para um pior prognóstico como uma das complicações mais prevalentes, afetando aproximadamente 60% dos pacientes em reabilitação pós-COVID.^6,7^ Em sobreviventes de casos críticos, a perda de peso média chega a 16% no momento da alta, um processo de depleção que pode levar meses para ser revertido, mesmo com acompanhamento especializado.^8^

Esse cenário é agravado pela sarcopenia aguda, identificada em quase metade dos pacientes na fase aguda e persistindo em cerca de 23% daqueles com COVID longa.^9^ Os cuidados em terapia intensiva para os pacientes com infecção pelo SARS-CoV-2 e suas complicações associadas podem levar à sarcopenia aguda, mesmo em indivíduos mais jovens.^10^ Os mecanismos são multifatoriais, incluindo o estado hipercatabólico, a inflamação sistêmica, a imobilização prolongada e a disfunção mitocondrial.^11,12^ Além disso, perdas sensoriais como a anosmia e a ageusia, aliadas à xerostomia e à disfagia, resultam em uma ingestão alimentar inadequada, exacerbando a degradação de miofibrilas e a proteólise muscular.^12^

O impacto dessa deterioração nutricional repercute diretamente no desempenho funcional discutido anteriormente. Estudos correlacionam baixos valores de ângulo de fase, um marcador de integridade celular, e redução na força de preensão manual com um pior prognóstico e maior dependência funcional.^6,8^ Curiosamente, enquanto a espessura e a força muscular podem melhorar com a reabilitação, o excesso de massa gordurosa acumulado pode influenciar negativamente o desempenho em testes de caminhada de seis minutos, indicando a necessidade de uma análise precisa da composição corporal, e não apenas do peso total.^13^

Diante desse panorama, o manejo da síndrome pós-COVID-19 exige que a reabilitação física seja indissociável do suporte nutricional agressivo. A implementação de dietas com maior aporte calórico e ricas em proteínas são fundamentais para romper o ciclo vicioso de desnutrição e declínio funcional. A monitoração regular da massa muscular, possivelmente por ultrassonografia à beira do leito, e a correção de deficiências de micronutrientes e aminoácidos essenciais tornam-se pilares indispensáveis para que as estratégias de exercício alcancem seu potencial máximo, devolvendo a autonomia e a qualidade de vida aos pacientes.^14^

Em conclusão, o manuscrito reforça a complexidade da síndrome pós-COVID-19, onde a rigidez arterial e o comprometimento funcional coexistem, embora nem sempre de forma linear. A sugestão de que o descondicionamento físico e a inflamação persistente podem ser os principais determinantes da limitação funcional aponta para a necessidade de estratégias de reabilitação. Como observado em estudos de intervenção, programas de exercícios supervisionados e treinamento de força muscular inspiratória mostram-se promissores para atenuar tanto a disfunção neurovascular quanto os sintomas de fadiga.
