# Supplementary material for: Rigidez Arterial e Comprometimento Funcional na Síndrome Pós-COVID-19: Um Enigma Multifatorial
Source: Arq Bras Cardiol. 2026 Jul 30;123(7):e20260519. [Article in Portuguese] doi: 10.36660/abc.20260519 (PMC13668447; doi:10.36660/abc.20260519)
Supplement: Translation

# Translation: Arterial Stiffness and Functional Impairment in Post-COVID-19 Syndrome: A Multifactorial Puzzle

**DOI:** 10.36660/abc.20260519i

**Authors:** Mario Fritsch Neves, Michelle Rabello Cunha

**Affiliations:** 1 Rio de Janeiro RJ Brazil Universidade do Estado do Rio de Janeiro, Rio de Janeiro, RJ – Brazil

**Keywords:** Vascular Stiffness, Post-Acute COVID-19 Syndrome

The cross-sectional study presented in the manuscript "Arterial Stiffness, Clinical-Functional Outcomes, and Quality of Life in Post-COVID-19 Patients: A Cross-Sectional Study" contributes to our understanding of the persistent sequelae of SARS-CoV-2 infection.^1^ The authors demonstrate that individuals in the post-COVID-19 period, compared to healthy controls, have higher pulse wave velocity (PWV) values, the primary marker of arterial stiffness. They also report a higher augmentation index (AIx@75), which does not directly indicate arterial stiffness but is a parameter reflecting pulse wave reflection; this can be influenced by arterial stiffness as well as by other confounding factors. These findings are consistent with global evidence, such as the multicenter CARTESIAN study, which identified early vascular aging in survivors of the disease, with a particularly pronounced impact on women.^2^

An intriguing aspect of the manuscript is the observation that, although PWV is elevated in the post-COVID group, the adjusted analysis suggests that this increase was predominantly driven by age and systolic blood pressure, rather than by the post-COVID-19 condition independently. On the other hand, some studies indicate that the deleterious effect of the virus on markers of arterial stiffness and endothelial function may persist for months after the acute phase has resolved.^3,4^

In addition to vascular changes, functional impairment and reduced quality of life emerge as hallmarks of post-COVID syndrome. The manuscript reports poorer performance on the sit-to-stand test, elevated levels of fatigue, and deterioration in physical and mental health. These findings are corroborated by research showing that even patients with mild to moderate cases experience clinically significant reductions in quality of life, often comparable to those with chronic obstructive pulmonary disease or fibromyalgia. They also highlight that the severity of fatigue is a key predictor of limitations in these individuals’ daily lives.^5^

The mechanism behind this functional decline appears to be multifactorial and not restricted solely to the macrovasculature. Muscle sympathetic hyperactivity and oxidative stress may persist for up to two years after infection.^3,4^ This autonomic hyperactivity, associated with microvascular dysfunction and persistent systemic inflammation, may impair oxygen delivery to skeletal muscles, explaining the exercise intolerance observed in this manuscript.

Post-COVID-19 syndrome is not merely a cardiovascular event, but a state of hypercatabolism and nutritional fragility that fuels a vicious cycle of functional decline. In addition to vascular and autonomic complexity, recent evidence highlights that functional impairment is profoundly influenced by changes in body composition and nutritional deficiencies. Malnutrition emerges both as a risk factor for a worse prognosis and as one of the most prevalent complications, affecting approximately 60% of patients undergoing post-COVID rehabilitation.^6,7^ In survivors of critical cases, the average weight loss reaches 16% at the time of discharge, a process of depletion that can take months to reverse, even with specialized care.^8^

This scenario is exacerbated by acute sarcopenia, identified in nearly half of patients during the acute phase and persisting in about 23% of those with long COVID.^9^ Intensive care for patients with SARS-CoV-2 infection and its associated complications can lead to acute sarcopenia, even in younger individuals.^10^ The mechanisms are multifactorial, including a hypercatabolic state, systemic inflammation, prolonged immobilization, and mitochondrial dysfunction.^11,12^ Furthermore, sensory losses such as anosmia and ageusia, combined with xerostomia and dysphagia, result in inadequate food intake, exacerbating myofibrillar degradation and muscle proteolysis.^12^

The impact of this nutritional deterioration directly affects the functional performance discussed earlier. Studies correlate low phase angle values, a marker of cellular integrity, and reduced handgrip strength with a worse prognosis and greater functional dependence.^6,8^ Interestingly, while muscle thickness and strength may improve with rehabilitation, accumulated excess fat mass can negatively influence performance on six-minute walk tests, indicating the need for an accurate analysis of body composition, rather than just total weight.^13^

Given this background, the management of post-COVID-19 syndrome requires that physical rehabilitation be inseparable from aggressive nutritional support. The implementation of high-calorie, protein-rich diets is essential to break the vicious cycle of malnutrition and functional decline. Regular monitoring of muscle mass, possibly via bedside ultrasound, and the correction of deficiencies in micronutrients and essential amino acids are indispensable pillars for exercise strategies to reach their full potential, restoring patients’ autonomy and quality of life.^14^

In conclusion, this manuscript underscores the complexity of post-COVID-19 syndrome, in which arterial stiffness and functional impairment coexist, though not always in a linear manner. Physical deconditioning and persistent inflammation may be the main determinants of functional limitation, pointing to the need for rehabilitation strategies. As observed in intervention studies, supervised exercise programs and inspiratory muscle strength training show promise in alleviating both neurovascular dysfunction and fatigue symptoms.
